# Phosphorus modifies the association between body mass index and uric acid: Results from NHANES 2007–2018

**DOI:** 10.1371/journal.pone.0306383

**Published:** 2024-10-10

**Authors:** Yue Chen, Jing Luo, Xiao-Man Ma, Xiang-Ping He, Wan-Lin Zhang, Shao-Yong Wu, Xiao-Chun Mo, Wei-Chao Huang, Xu-Guang Guo

**Affiliations:** 1 Department of Clinical Laboratory Medicine, Guangdong Provincial Key Laboratory of Major Obstetric Diseases, Guangzhou, China; 2 Guangdong Provincial Clinical Research Center for Obstetrics and Gynecology, Guangzhou, China; 3 The Third Affiliated Hospital of Guangzhou Medical University, Guangzhou, China; 4 Department of Anesthesiology, The Second Clinical School of Guangzhou Medical University, Guangzhou, China; 5 Department of Clinical Medicine, The Third Clinical School of Guangzhou Medical University, Guangzhou, China; 6 Department of Clinical Medicine, The Chinese and Western Clinical School of Guangzhou Medical University, Guangzhou, China; 7 Department of Clinical Medicine, The First Clinical School of Guangzhou Medical University, Guangzhou, China; 8 Department of Clinical Medicine, The Second Clinical School of Guangzhou Medical University, Guangzhou, China; 9 Guangzhou Key Laboratory for Clinical Rapid Diagnosis and Early Warning of Infectious Diseases, King Med School of Laboratory Medicine, Guangzhou Medical University, Guangzhou, China; Istituto Di Ricerche Farmacologiche Mario Negri, ITALY

## Abstract

**Introduction:**

Studies in recent years have shown that high uric acid causes harm to the human body, which has become a serious public health problem. Elevated serum uric acid has been shown to be associated with obesity, but the relationship between BMI and uric acid (UA) remains controversial. Although the association between BMI and UA has been well studied, the effect of phosphorus levels in vivo on this association remains unclear. This study aimed to determine the relationship between BMI and serum uric acid and the effect of phosphorus on the relationship between the two.

**Research design and methods:**

The present study analyzed data from the National Health and Nutrition Examination Survey (NHANES) continuous 2007–2018 cycle. We included 10786 participants aged 20 years and over. Multivariable linear regression was performed to assess the association between BMI and serum uric acid. phosphorus was stratified into low phosphorus (<3.3 mg/dl), middle phosphorus (3.3–3.9 mg/dl) and high phosphorus (>3.9 mg/dl). Correction of the effect of phosphorus was assessed by testing the interaction between BMI and UA in multivariate linear regression.

**Results:**

In this cross-sectional study, we found that BMI was positively associated with UA in the female population but not significantly in the male population or in the total population. In multiple regression analysis, UA was 0.51 higher in the highest female BMI group than in the lowest group (p = 0.0001). The relationship between BMI and UA differed significantly by gender under the influence of phosphorus, with men and women in Model II having a greater elevation of UA in men than in women within most groups. (BMI >30, phosphorus >3.9 mg/dl, β:0.83 95% CI: 0.43, 1.23 vs β: 0.79 95% CI: 0.30, 1.29). In addition, phosphorus significantly altered the positive association between BMI and UA in most models.

**Conclusion:**

Our results indicate significant associations between BMI and uric acid in women, with higher BMI values likely to be associated with a higher risk of hyperuricemia, suggesting that uric acid levels in obese people should be closely monitored in clinical practice. Phosphorus and BMI have an interactive effect in elevating UA and should be noted as indicators of phosphorus in clinical practice.

## Introduction

In recent decades, many studies have found that high uric acid levels cause an unhealthy body and serious harm, and hyperuricemia has become an increasingly serious public health problem [1, 2]. A review conducted by Li Q showed that the prevalence of hyperuricemia is approximately 13.3% in hypertensive patients [3]. Uric acid (UA) is primarily a major oxidation product of purine metabolism, which generates harmful effects in vascular smooth muscle cells and fat cells, such as inhibition of endothelial function and induction of platelet aggregation [4, 5]. Hyperuricemia is strongly associated with gout, chronic kidney disease, stroke and cardiovascular disease [3, 6–8]. Therefore, it is important for us to correctly understand hyperuricemia and the range of diseases it can lead to. Some cross-sectional studies have shown that factors associated with elevated uric acid closely related to general obesity defined by body mass index (BMI) [3, 6].

Regarding the relationship between BMI and uric acid, some studies have found a positive correlation between BMI and uric acid [9–12], which may be affected by gender [13, 14], while others have shown no correlation [15, 16]. Most of the epidemiological data on obesity are based on BMI (kg/m2) [17]. The range of 18.5–25 is normal, ≥25 is overweight, and ≥30 is obese [18]. Overweight as a preceding stage of obesity also has important clinical significance. Obesity is a basic risk factor for the onset and development of insulin resistance, and obese people tend to have insulin resistance [19], which is a reason for high serum uric acid levels in obese patients [20]. In addition, the increase in visceral fat in obese patients is also a reason for the increase in serum uric acid levels [21]. These mechanisms may explain the relationship between obesity and high serum uric acid. Based on the above findings, we hypothesized that BMI was significantly correlated with uric acid.

Phosphorus is a major element in the human body and plays an important role in cell metabolism and tissue structure [22]. One longitudinal multicenter study found that the BMI level of patients with hypophosphatemia was low [23], but another study found that phosphorus was negatively correlated with BMI [24]. phosphorus also affects the metabolism of uric acid. Studies have shown that patients with hyperuricemia have higher serum uric acid levels [25]. Therefore, in this study, we hypothesized that magnesium interacts with BMI on serum uric acid. We used data from the NHANES to better understand the relationship between BMI and SUA and the effect of phosphorus on the relationship between the two.

## Methods

### Study design and population

NHANES is a national representative cross-sectional survey of the noninstitutionalized civilian population in the United States, conducted annually by the Centers for Disease Control and Prevention’s National Center for Health Statistics (CDC/NCHS). This survey conducted a household interview and a physical examination in a mobile examination center (MEC). The NHANES interview included questions about demographics, socioeconomics, diet and health. Examination parts included medical, dental and physiological measurements and clinical examinations by trained medical staff. In 1999, the survey became a continuous program and data were released to the public in two-year cycles, which has a changing focus on a variety of health and nutrition measurements to meet emerging needs.

This cross-sectional study uses data from the NHANES 2007–2008, 2009–2010, 2011–2012, 2013–2014, 2015–2016 and 2017–2018 cycles. All procedures were approved by the NCHS Research Ethics Review Board (https://www.cdc.gov/nchs/nhanes/irba98.htm), and all participants provided written informed consent. We enrolled 59744 participants who completed the interview, and 34770 adults (≥20 years old) were enrolled in the study. Participants whose data were missing on BMI (n = 1831), serum uric acid (n = 2064) and covariates (n = 20089) were excluded. We ultimately included and analyzed 10786 participants in total.

### Measurement of SUA

Serum samples for measurement of the study population collected during the MEC examination were processed and stored at -30°C until shipped to CDC/NCEH/DLS for testing. The concentration of SUA was measured as part of routine serum biochemical profiling using the Beckman Coulter UniCel^®^DxC800 with a timed endpoint method (https://wwwn.cdc.gov/nchs/data/nhanes/2013-2014/labmethods/BIOPRO_H_MET_URIC_ACID.pdf).

### Measurement of BMI

BMI was calculated by dividing weight (kg) by the square of height (m). The height of the subject was measured by a standard stadiometer, while the weight was measured by an electronic balance [26]. According to WHO standards, BMI of adults was divided into underweight (BMI<18.5kg/m^2^), normal weight (18.5kg/m^2^≤BMI<25kg/m^2^), and obese (25kg/m^2^≤BMI<30kg/m^2^) and obesity (BMI≥30 kg/m^2^) [18].

### Measurement of phosphorus

The DxC800 system is used to determine phosphorus concentrations in serum, plasma and urine using the timed rate method. In the reaction, inorganic phosphorus reacts with ammonium molybdate in an acidic solution to form colored phosphomolybdate complexes. This change in absorbance is proportional to the concentration of phosphorus in the sample. phosphorus levels were measured by monitoring the change in absorbance at 365 nm at fixed time intervals.

### Covariates

The participants’ demographic and lifestyle information was collected in a mobile examination center (MEC). The present study considers age, BMI, ucid acid, eGFR, triglycerides, creatinine, glucose, plasma glucose, total cholesterol, direct hdl-cholesterol, ldl-cholesterol, glycohemoglobin, Waist circumference, DBP(diastolic blood pressure), SBP(systolic blood pressure), minutes sedentary activity, sex, race, education level, marital status, ratio of family income to poverty, work activity, recreational activity, hypertension, DM(diabetes mellitus), smoking status, alcohol consumption level, doctor ever said you had arthritis Race/ethnicity was categorized as Mexican American, Other Hispanic, Non-Hispanic White, Non-Hispanic Black and Other Races. Smoking status is divided into current smoker (who have smoked more than 100 cigarettes in a lifetime and currently smoke), former smoker (who have smoked more than 100 cigarettes in a lifetime but have not smoked) and never smoker (who have never smoked more than 100 cigarettes). Datas on alcohol drinking consumption were obtained by questionnaire interviews, which is divided to never drinking, former drinking, mild drinking, moderate drinking and heavy drinking. Educational levels were categorized as did not graduate from high school, graduated from high school, college education or above. Blood pressure measurements were obtained by a trained physician manually auscultating with a mercury gravimeter using a standardized protocol. Respondents were classified as having high blood pressure if they answered "yes" to the following question: "Has a doctor or other health professional ever told you that you have high blood pressure or so-called hypertension?" DM was categorized as No (which was defined as having FPG < 5.6 mmol/l (<100 mg/dl) and 2hPG < 7.8 mmol/l (<140 mg/dl)), DM(which was defined as having FPG ≥ 7.0 mmol/l (≥126 mg/dl) and/or 2hPG ≥ 11.1 mmol/l (≥200 mg/dl)), IFG (which was defined as having FPG ≥5.6 mmol/l (100 mg/dl) but <7.0 mmol/l (126 mg/dl)) and IGT(which was defined as having 2-h glucose ≥7.8 mmol/l (140 mg/dl) but <11.1 mmol/l (200 mg/dl)) [27]. Respondents were classified as having gout if they answered "yes" to the question: "Has a doctor or other health professional ever told you that you have gout?" In this study, if the SUA level of men was 7 mg/dL and that of women was > 6 mg/dL, it was defined as hyperuricemia [24]. Specific information concerning the serum contents of triglycerides, creatinine, and total cholesterol was extracted from the NHANES laboratory detection data. The formula for the estimated glomerular filtration rate was as follows:$\text{eGFR}=175\times {\left(\frac{creatinine}{88.4}\right)}^{-1.234}\times age^{-0.179}\times (0.79\;for\;females)$.

### Statistical analyses

All the statistical analyses were performed using EmpowerStats (www.empowerstats.com, X&Y solution, Inc. Boston MA) and R software version 3.6.1(http://www.r-ptoject.org). To examine the association between BMI and uric acid, multivariate linear regression procedures were performed. To ensure the accuracy of the conclusion, we adjusted for sex, age, race, education level, smoking, alcohol use, magnesium, dietary fiber, total sugars, vitamin D (D2+D3), vitamin C, energy, triglyceride, creatinine, diastolic blood pressure (DBP), systolic blood pressure (SBP), total cholesterol, estimated glomerular filtration rate, diagnosed with high blood pressure, and gout, among different ranges of BMI. Ninety-five percent confidence intervals (CIs) were calculated. To observe the internal relationship between BMI and SUA, we used smooth curve fitting. Stratified regression analyses were used to account for differences in phosphorus. Statistical tests with p values<0.05 were considered significant. Continuous variables are presented as the mean and standard deviation (SD) or median and interquartile range (IQR), and categorical variables are presented as weighted percentages (%) in descriptive analysis. At the same time, the chi-square test (categorical variables) and Kruskal‒Wallis test (skewness distribution) were performed to make statistical inferences for continuous variables and categorical variables. Standardized beta was utilized to compare the relative predictive strength of different covariates in the regression models. The variance inflation factor (VIF) was used to assess the multicollinearity of all covariates in the regression model.

### Ethics approval and consent to participate

The NHANES survey protocol was approved by the Research Ethics Review Board of the National Institutes of Health Statistics, a division of the Centers for Disease Control and Prevention. All participants submitted written informed consent and were approved by the NCHS Research Ethics Review Board (https://wwwn.cdc.gov/nchs/nhanes/default.aspx).

## Results

### Baseline characteristics of the study participants

**Fig 1:**
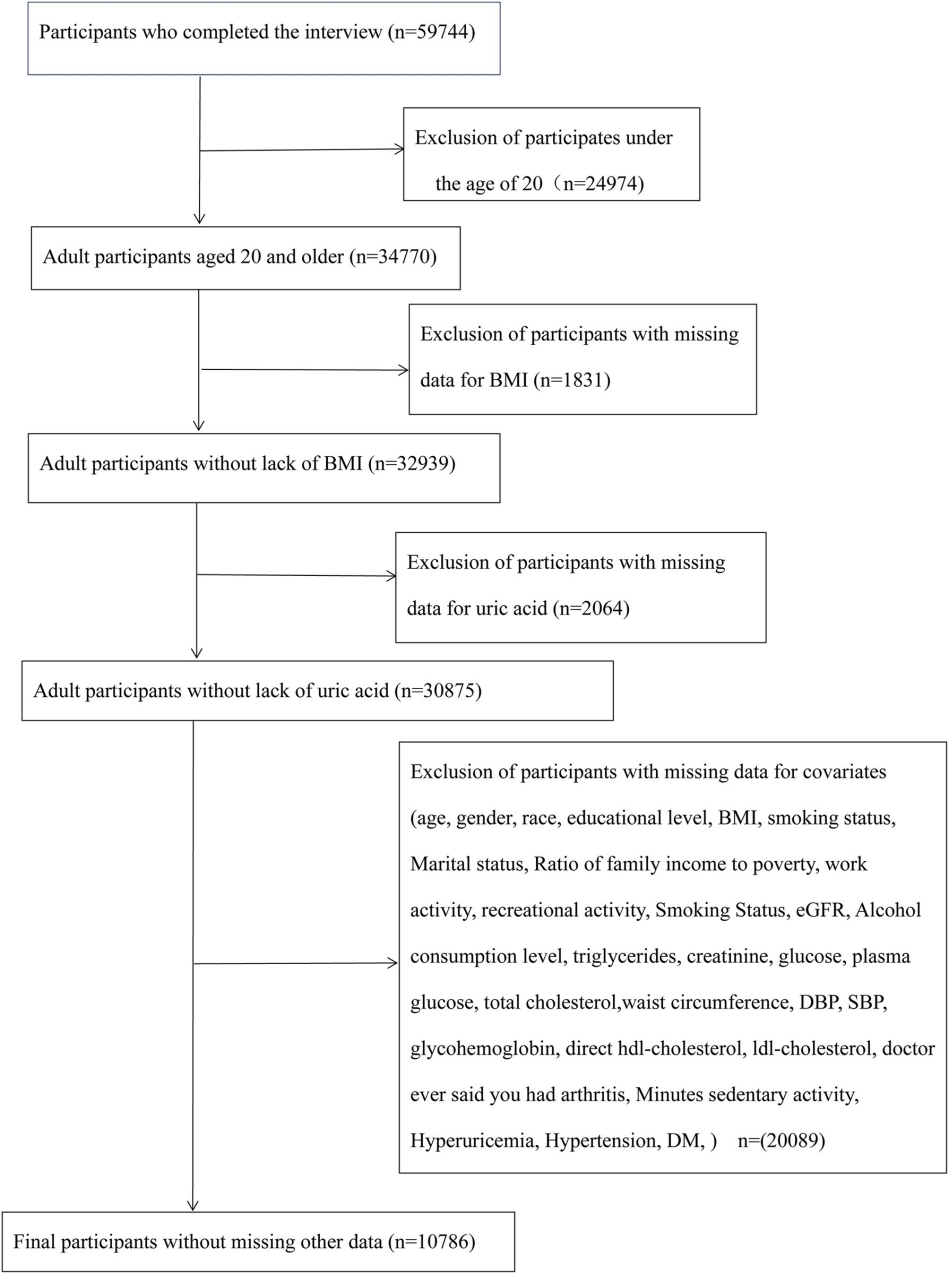
Flow chart for inclusion and exclusion of participants.

The six cycles of NHANES 2007–2018 were used in our current study. We identified 59,744 participants in the study who had recently completed an interview and MEC assessment. Subsequently, 34770 participants aged ≥20 years were included as adults. Participants with missing data for BMI (n = 1831) and uric acid (UA) (n = 2064) were excluded. After excluding participants with missing data such as other covariates, our analysis included 10786 participants. A flow chart of the exclusion criteria is shown in Fig 1.

The baseline characteristics of the participants are shown in Table 1. The population was divided into four categories based on BMI values, with BMI <18.5 being the low BMI group (Q1), BMI >18.5, < = 25 being the normal BMI group (Q2), BMI >25, < = 30 being the overweight group (Q3), and BMI >30 being the obese group (Q4). Compare with the Q1 group (BMI<18.5), the participants in Q4 group (BMI>30) was more likely to be older, male, less non-Hispanic White, less other race, and now smoke, have no diabetes, live alone, have vigorous recreational activities, never drink alcohol, and the proportion of direct high-density lipoprotein cholesterol is low. The Q4 group had a higher rate of hypertension, higher concentrations of serum uric acid, triglycerides, creatinine, glucose, plasma glucose, total cholesterol, LDL-cholesterol, glycohemoglobin, waist circumference, DBP, SBP, a lower ratio of family income to poverty and lower estimated glomerular filtration rates. No statistically significant differences were found in minutes of sedentary activity, education level or work activity (P >0.05).

**Table 1:** Baseline characteristics of participants (N = 10786).

Characteristic	BMI (kg/m^2)				P-value
	Q1 < = 18.5	Q2 > 18.5, < = 25	Q3 > 25, < = 30	Q4 > 30	
N					
Age (year)	41.73 (38.48, 44.97)	44.93 (43.87, 46.00)	49.07 (48.35, 49.79)	48.69 (47.89, 49.49)	<0.0001
BMI(kg/m^2)	17.71 (17.59, 17.83)	22.47 (22.37, 22.56)	27.44 (27.37, 27.51)	35.91 (35.66, 36.16)	<0.0001
uric acid (mg/dl)	4.42 (4.22, 4.61)	4.95 (4.89, 5.01)	5.52 (5.46, 5.57)	5.94 (5.88, 5.99)	<0.0001
eGFR (ml/min/1.73m^2^)	104.24 (100.41, 108.07)	97.38 (96.20, 98.56)	93.07 (92.09, 94.05)	94.40 (93.37, 95.44)	<0.0001
triglycerides (mg/dl)	84.91 (74.68, 95.14)	93.83 (91.46, 96.20)	124.93(121.67, 128.19)	140.93 (136.70, 145.17)	<0.0001
creatinine (umol/l)	72.11 (67.14, 77.08)	76.34 (74.94, 77.73)	78.94 (78.11, 79.76)	77.17 (76.10, 78.24)	0.0008
glucose (mg/dl)	89.65 (87.28, 92.02)	92.11 (91.17, 93.05)	98.12 (97.21, 99.02)	108.07 (106.43, 109.70)	<0.0001
plasma glucose(mg/dl)	96.47 (93.95, 99.00)	98.71 (97.74, 99.68)	104.67 (103.73, 105.60)	114.26 (112.60, 115.92)	<0.0001
total cholesterol (mmol/l)	4.73 (4.51, 4.95)	4.86 (4.81, 4.91)	5.06 (5.02, 5.11)	4.96 (4.91, 5.01)	<0.0001
direct hdl-cholesterol (mg/dl)	66.92 (63.24, 70.59)	61.67 (60.76, 62.57)	53.63 (52.81, 54.46)	48.87 (48.32, 49.41)	<0.0001
ldl-cholesterol (mg/dl)	100.21 (93.57, 106.86)	107.97 (106.27, 109.68)	118.06 (116.48, 119.63)	116.06 (114.60, 117.52)	<0.0001
glycohemoglobin(%)	5.32 (5.25, 5.39)	5.41 (5.38, 5.44)	5.55 (5.52, 5.58)	5.87 (5.82, 5.92)	<0.0001
Waist circumference (cm)	71.59 (70.82, 72.36)	83.49 (83.13, 83.85)	97.04 (96.71, 97.37)	115.15 (114.56, 115.74)	<0.0001
DBP(mmHg)	65.22 (63.31, 67.13)	67.30 (66.73, 67.88)	69.46 (68.79, 70.13)	71.42 (70.77, 72.06)	<0.0001
SBP(mmHg)	112.16 (109.35, 114.97)	116.71 (115.93, 117.49)	120.64 (119.96, 121.33)	124.11 (123.42, 124.79)	<0.0001
minutes sedentary activity (min)	361.04 (316.71, 405.36)	381.97 (357.28, 406.65)	382.94 (368.49, 397.38)	396.28 (386.53, 406.03)	0.2960
Sex					<0.0001
male	36.81 (28.93, 45.47)	44.81 (42.42, 47.22)	57.37 (55.36, 59.36)	47.84 (45.84, 49.85)	
female	63.19 (54.53, 71.07)	55.19 (52.78, 57.58)	42.63 (40.64, 44.64)	52.16 (50.15, 54.16)	
Race					<0.0001
Mexican American	3.49 (1.66, 7.22)	5.14 (4.23, 6.24)	8.84 (7.33, 10.63)	10.01 (8.13, 12.26)	
Other Hispanic	3.28 (1.56, 6.76)	4.55 (3.56, 5.81)	5.91 (4.80, 7.27)	5.53 (4.45, 6.86)	
Non-Hispanic White	68.31 (59.23, 76.18)	71.40 (68.36, 74.26)	70.91 (67.93, 73.73)	67.56 (63.78, 71.13)	
Non-Hispanic Black	8.36 (5.25, 13.07)	7.84 (6.67, 9.19)	8.23 (7.09, 9.53)	12.48 (10.52, 14.74)	
Other Race—Including Multi-Racial	16.55 (11.32, 23.56)	11.07 (9.48, 12.89)	6.11 (5.00, 7.44)	4.42 (3.55, 5.48)	
Education level					0.1268
≤High school	22.29 (14.65, 32.40)	20.32 (17.71, 23.20)	23.09 (20.60, 25.78)	23.38 (21.21, 25.70)	
>High school	77.71 (67.60, 85.35)	79.68 (76.80, 82.29)	76.91 (74.22, 79.40)	76.62 (74.30, 78.79)	
Marital status					<0.0001
Married	45.36 (36.87, 54.14)	52.81 (49.50, 56.09)	58.46 (55.88, 61.00)	57.41 (54.68, 60.09)	
Widowed	4.59 (1.71, 11.78)	4.64 (3.87, 5.54)	5.38 (4.51, 6.41)	5.52 (4.66, 6.51)	
Divorced	8.09 (4.38, 14.47)	9.09 (7.58, 10.87)	11.35 (10.11, 12.72)	11.09 (9.66, 12.70)	
Separated	0.48 (0.11, 2.20)	2.41 (1.86, 3.13)	1.74 (1.39, 2.19)	2.42 (1.85, 3.17)	
Never married	28.29 (21.20, 36.64)	22.93 (20.64, 25.38)	14.87 (13.26, 16.64)	15.97 (14.18, 17.93)	
Living with partner	13.18 (7.23, 22.84)	8.13 (6.77, 9.72)	8.19 (6.89, 9.73)	7.59 (6.50, 8.85)	
Ratio of family income to poverty					0.0001
< = 1	21.24 (14.47, 30.07)	13.91 (12.22, 15.79)	12.71 (11.36, 14.19)	14.64 (12.91, 16.55)	
1–3	37.40 (28.45, 47.30)	32.92 (30.24, 35.72)	34.05 (31.65, 36.53)	37.98 (35.70, 40.31)	
≥3	41.35 (31.19, 52.31)	53.17 (49.71, 56.61)	53.24 (50.45, 56.02)	47.38 (44.51, 50.28)	
Work activity					0.0631
no	55.84 (47.63, 63.73)	53.48 (50.57, 56.36)	52.57 (50.13, 54.99)	52.62 (50.31, 54.91)	
both	13.82 (9.09, 20.47)	17.81 (15.76, 20.05)	19.79 (17.90, 21.82)	17.23 (15.51, 19.09)	
moderate	25.73 (17.79, 35.67)	25.83 (23.59, 28.20)	23.21 (21.21, 25.34)	25.52 (23.62, 27.52)	
vigorous	4.61 (1.24, 15.66)	2.89 (2.26, 3.68)	4.44 (3.58, 5.48)	4.63 (3.82, 5.60)	
Recreational activity					<0.0001
no	47.28 (36.59, 58.22)	36.19 (33.05, 39.44)	42.88 (40.42, 45.39)	53.90 (51.44, 56.35)	
both	20.68 (13.05, 31.18)	24.04 (21.57, 26.69)	18.94 (17.26, 20.74)	11.91 (10.42, 13.58)	
moderate	24.08 (16.33, 34.02)	28.74 (26.25, 31.37)	28.90 (26.68, 31.24)	28.44 (26.53, 30.42)	
vigorous	7.96 (3.79, 15.94)	11.04 (9.51, 12.78)	9.28 (8.07, 10.64)	5.75 (4.87, 6.78)	
Hypertension					<0.0001
No	81.72 (72.82, 88.18)	77.15 (74.79, 79.36)	63.27 (61.20, 65.30)	48.53 (46.34, 50.72)	
Yes	18.28 (11.82, 27.18)	22.85 (20.64, 25.21)	36.73 (34.70, 38.80)	51.47 (49.28, 53.66)	
DM					<0.0001
No	85.30 (76.57, 91.16)	82.08 (79.99, 83.99)	68.61 (66.51, 70.64)	52.14 (49.88, 54.39)	
DM	4.49 (2.59, 7.68)	6.75 (5.69, 7.99)	13.05 (11.78, 14.43)	25.74 (23.73, 27.85)	
IGT	6.33 (3.09, 12.53)	5.90 (4.94, 7.03)	7.44 (6.39, 8.64)	8.08 (7.07, 9.23)	
IFG	3.87 (1.09, 12.88)	5.27 (4.28, 6.48)	10.91 (9.65, 12.30)	14.05 (12.29, 16.00)	
Smoking Status					<0.0001
never	51.94 (42.04, 61.69)	56.03 (53.03, 59.00)	54.14 (51.80, 56.47)	55.47 (53.22, 57.69)	
now	33.51 (23.68, 45.02)	23.73 (21.06, 26.63)	17.75 (16.05, 19.59)	15.97 (14.47, 17.59)	
former	14.55 (8.84, 23.03)	20.23 (18.16, 22.48)	28.10 (25.86, 30.46)	28.56 (26.47, 30.75)	
Alcohol consumption level					<0.0001
never	13.94 (9.26, 20.45)	10.13 (8.76, 11.67)	8.68 (7.53, 10.00)	10.98 (9.63, 12.50)	
former	12.35 (6.87, 21.22)	9.00 (7.61, 10.61)	12.62 (11.14, 14.26)	15.21 (13.64, 16.92)	
Mild	31.72 (22.58, 42.54)	40.61 (37.74, 43.55)	40.59 (38.09, 43.13)	35.43 (33.23, 37.69)	
Moderate	19.63 (12.54, 29.39)	18.58 (16.65, 20.69)	17.43 (15.73, 19.27)	17.25 (15.66, 18.96)	
Heavy	22.35 (14.43, 32.96)	21.68 (19.53, 23.99)	20.69 (18.95, 22.54)	21.13 (19.21, 23.19)	
doctor ever said you had arthritis					<0.0001
No	98.45 (95.86, 99.43)	98.33 (97.77, 98.75)	96.50 (95.69, 97.16)	93.88 (92.79, 94.82)	
Yes	1.55 (0.57, 4.14)	1.67 (1.25, 2.23)	3.50 (2.84, 4.31)	6.12 (5.18, 7.21)	

Abbreviations: BMI body mass index, SBP systolic blood pressure, DBP diastolic blood pressure, eGFR glomerular filtration rate

*BMI was calculated as the body weight in kilograms divided by the square of the height in meters

Data in Table 1, for continuous variables: survey-weighted mean (95% CI), P value was by survey-weighted linear regression (svyglm); for categorical variables: survey-weighted percentage (95% CI), P value was by survey-weighted Chi-square test (svytable)

The population was divided into two categories based on hyperuricemia and the baseline characteristics of the participants are shown in Table 2. Men with uric acid >7 mg/dl and women with uric acid >6 mg/dl were defined as hyperuricemic. Those with hyperuricemia were more likely to be older men with higher BMI, triglycerides, creatinine, glucose, glycated hemoglobin, plasma glucose, total cholesterol, LDL-cholesterol, waist circumference, DBP, SBP, sedentary activity time, a higher proportion of non-Hispanic whites, non-Hispanic blacks and other races, living alone, no or moderate recreational activity, and former smokers with higher rates of hypertension, DM, IGT, IFG, and arthritis. No statistically significant differences were found in education level, household income to poverty ratio, work activity or alcohol consumption levels (p>0.05).

**Table 2:** Baseline characteristics of participants on hyperuricemia. (N = 10786).

Characteristic	Hyperuricemia		P value
	No	Yes	
N	8722	2064	
Age (year)	46.86 (46.23, 47.49)	51.07 (50.03, 52.12)	<0.0001
BMI (kg/m^2)	28.14 (27.91, 28.36)	32.49 (32.07, 32.91)	<0.0001
uric acid (mg/dl)	5.05 (5.02, 5.08)	7.47 (7.42, 7.51)	<0.0001
eGFR (ml/min/1.73m^2^)	97.22 (96.46, 97.98)	84.89 (83.52, 86.25)	<0.0001
triglycerides (mg/dl)	114.78 (112.64, 116.92)	149.93 (144.37, 155.49)	<0.0001
creatinine (umol/l)	75.16 (74.49, 75.82)	87.67 (86.19, 89.15)	<0.0001
glucose (mg/dl)	98.95 (98.08, 99.82)	104.01 (102.34, 105.68)	<0.0001
glycohemoglobin(%)	5.59 (5.56, 5.62)	5.75 (5.70, 5.81)	<0.0001
plasma glucose(mg/dl)	105.51 (104.57, 106.45)	109.92 (108.28, 111.55)	<0.0001
total cholesterol (mmol/l)	4.93 (4.90, 4.96)	5.09 (5.02, 5.15)	<0.0001
direct hdl-cholesterol (mg/dl)	55.38 (54.84, 55.91)	50.11 (48.98, 51.25)	<0.0001
ldl-cholesterol (mg/dl)	113.28 (112.37, 114.19)	118.10 (115.78, 120.42)	0.0001
Waist circumference (cm)	97.26 (96.71, 97.81)	108.58 (107.54, 109.61)	<0.0001
DBP(mmHg)	69.27 (68.76, 69.78)	70.44 (69.59, 71.29)	0.0059
SBP(mmHg)	119.72 (119.17, 120.26)	124.78 (123.68, 125.87)	<0.0001
minutes sedentary activity (min)	383.12 (370.78, 395.47)	405.18 (388.34, 422.02)	0.0392
Sex			<0.0001
male	48.58 (47.31, 49.85)	56.29 (53.63, 58.91)	
female	51.42 (50.15, 52.69)	43.71 (41.09, 46.37)	
Race			<0.0001
Mexican American	8.59 (7.20, 10.23)	5.99 (4.64, 7.70)	
Other Hispanic	5.62 (4.62, 6.83)	4.10 (3.13, 5.35)	
Non-Hispanic White	69.41 (66.56, 72.11)	71.53 (67.77, 75.01)	
Non-Hispanic Black	9.35 (8.11, 10.75)	11.07 (9.21, 13.27)	
Other Race—Including Multi-Racial	7.03 (6.21, 7.95)	7.30 (5.89, 9.03)	
Education level			0.7774
≤High school	22.33 (20.36, 24.43)	22.65 (20.09, 25.42)	
>High school	77.67 (75.57, 79.64)	77.35 (74.58, 79.91)	
Marital status			<0.0001
Married	56.09 (54.10, 58.06)	56.97 (53.85, 60.03)	
Widowed	4.52 (3.97, 5.15)	8.25 (6.99, 9.73)	
Divorced	10.51 (9.61, 11.49)	10.75 (8.96, 12.84)	
Separated	2.23 (1.84, 2.69)	1.86 (1.33, 2.61)	
Never married	18.21 (16.76, 19.76)	15.91 (13.80, 18.28)	
Living with partner	8.44 (7.50, 9.48)	6.25 (5.06, 7.70)	
Ratio of family income to poverty			0.1495
< = 1	14.23 (12.85, 15.72)	12.39 (10.84, 14.12)	
1–3	34.92 (33.15, 36.73)	36.50 (33.29, 39.83)	
≥3	50.85 (48.44, 53.27)	51.12 (47.52, 54.71)	
Work activity			0.8009
no	52.70 (50.99, 54.40)	53.80 (50.17, 57.39)	
both	18.14 (16.95, 19.40)	18.43 (15.71, 21.50)	
moderate	24.98 (23.60, 26.41)	24.20 (21.32, 27.32)	
vigorous	4.18 (3.58, 4.87)	3.57 (2.56, 4.98)	
Recreational activity			<0.0001
no	43.91 (41.74, 46.11)	50.09 (47.11, 53.07)	
both	18.59 (17.21, 20.05)	14.65 (12.63, 16.92)	
moderate	28.32 (26.69, 30.00)	29.91 (27.57, 32.36)	
vigorous	9.18 (8.35, 10.07)	5.35 (4.29, 6.65)	
Hypertension			<0.0001
No	66.70 (65.01, 68.35)	41.96 (38.92, 45.07)	
Yes	33.30 (31.65, 34.99)	58.04 (54.93, 61.08)	
DM			<0.0001
No	70.13 (68.45, 71.76)	51.61 (48.86, 54.34)	
DM	14.00 (12.91, 15.17)	23.39 (20.69, 26.32)	
IGT	6.76 (6.08, 7.50)	9.25 (7.61, 11.21)	
IFG	9.11 (8.18, 10.14)	15.75 (13.48, 18.32)	
Smoking Status			<0.0001
never	55.87 (53.98, 57.74)	51.80 (48.87, 54.71)	
now	19.68 (18.16, 21.30)	16.34 (14.00, 18.99)	
former	24.45 (22.89, 26.07)	31.86 (28.71, 35.19)	
Alcohol consumption level			0.0568
never	10.17 (9.00, 11.48)	9.31 (7.85, 11.02)	
former	12.04 (11.03, 13.13)	14.68 (12.78, 16.81)	
Mild	38.93 (36.97, 40.92)	37.01 (33.89, 40.25)	
Moderate	17.99 (16.85, 19.19)	16.55 (14.22, 19.18)	
Heavy	20.87 (19.52, 22.29)	22.44 (20.24, 24.81)	
doctor ever said you had arthritis			<0.0001
No	97.41 (96.94, 97.80)	90.26 (88.46, 91.80)	
Yes	2.59 (2.20, 3.06)	9.74 (8.20, 11.54)	

Abbreviations: BMI body mass index, SBP systolic blood pressure, DBP diastolic blood pressure, eGFR glomerular filtration rate

Hyperuricemia (diagnosis based on uric acid value): male > 7 mg/dl, female > 6 mg/dl

*BMI was calculated as the body weight in kilograms divided by the square of the height in meters

Data in Table 1, for continuous variables: survey-weighted mean (95% CI), P value was by survey-weighted linear regression (svyglm); for categorical variables: survey-weighted percentage (95% CI), P value was by survey-weighted Chi-square test (svytable)

### Analysis of the nonlinear relationship

**Fig 2:**
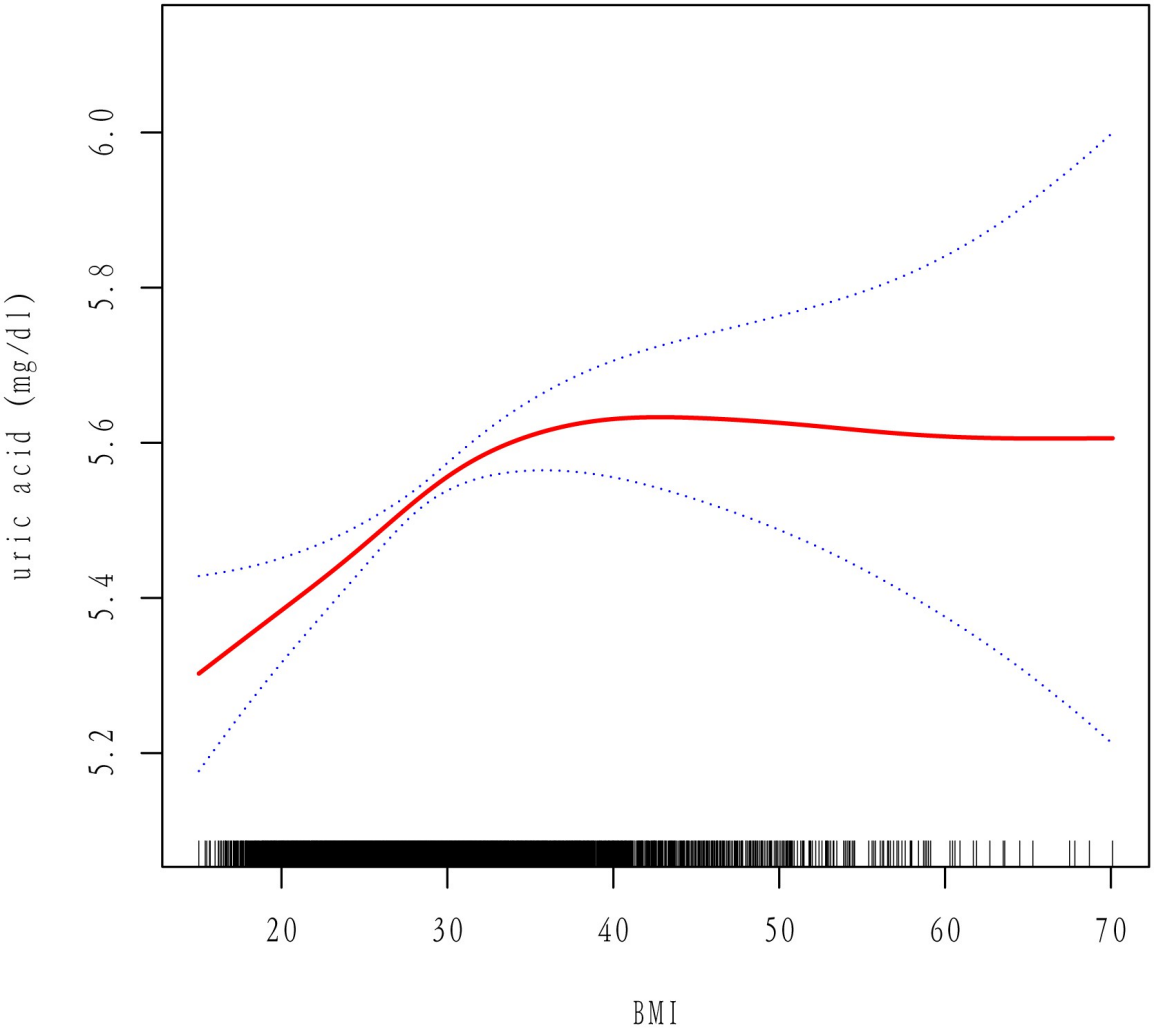
Association of BMI with uric acid (UA), adjusted by model 2.

We selected these confounders based on their association with the outcome of interest or a change in effect estimate of more than 10%. S1 Table shows the association of each confounder with the outcome of interest. As BMI is a continuous variable, it was necessary to analyze its linear relationship. The curve fitting results shown in Fig 2 indicate a **nonlinear** relationship between BMI and UA.

In addition, we performed a threshold effects model analysis (S2 Table) and based on the log-likelihood ratio test (p<0.001), we concluded that Model II better reflected the relationship between BMI and UA and was described as nonlinear. The inflection point in the fully adjusted variable model was 30.54 for the male population, 34.3 for the female population and 30.8 for the total population. Prior to the inflection point, BMI and UA were positively correlated in all populations.

### Association between BMI and uric acid

According to the multivariate linear regression results shown in Table 3, a weak but significant association was found with serum uric acid when BMI was a continuous variable (p<0.05) for men (β: 0.01, CI: 0.00, 0.02) and women (β: 0.02, CI: 0.01, 0.03). When grouping BMI, the low BMI group (BMI <18.5) was used as a reference. In women, a positive association between BMI and uric acid was significant in all models. In the fully adjusted model (model 2), UA concentration levels increased by 0.51 mg/dl (P = 0.0001) for every 1 unit increase in the female obese group (BMI >30) and by 0.23 mg/dl (P = 0.0320) in the female normal BMI group (BMI >18.5, < = 25). In men, BMI was significantly associated with uric acid only in the unadjusted model.

**Table 3:** Association between BMI and uric acid (UA).

	Male		Female		Total	
Outcome	β(95%Cl)	P value	β(95%Cl)	P value	β(95%Cl)	P value
Crude Model						
BMI	0.06 (0.06, 0.07)	<0.0001	0.06 (0.06, 0.07)	<0.0001	0.06 (0.06, 0.07)	<0.0001
BMI categorical						
< = 18.5	Reference (0)		Reference (0)		Reference (0)	
>18.5, < = 25	0.37 (0.10, 0.65)	0.0098	0.47 (0.27, 0.67)	<0.0001	0.53 (0.33, 0.73)	<0.0001
>25, < = 30	0.79 (0.51, 1.07)	<0.0001	0.88 (0.65, 1.10)	<0.0001	1.10 (0.90, 1.31)	<0.0001
>30	1.25 (0.98, 1.52)	<0.0001	1.49 (1.27, 1.70)	<0.0001	1.52 (1.34, 1.71)	<0.0001
Model 1						
BMI	0.01 (0.00, 0.02)	0.0268	0.02 (0.01, 0.03)	<0.0001	-0.06 (-0.07, -0.05)	<0.0001
BMI categorical						
< = 18.5	Reference (0)		Reference (0)		Reference (0)	
>18.5, < = 25	0.07 (-0.17, 0.31)	0.5648	0.25 (0.05, 0.44)	0.0159	0.10 (-0.09, 0.28)	0.3083
>25, < = 30	0.13 (-0.13, 0.39)	0.3287	0.40 (0.19, 0.61)	0.0004	0.13 (-0.07, 0.33)	0.2056
>30	0.19 (-0.08, 0.47)	0.1738	0.55 (0.33, 0.78)	<0.0001	-0.09 (-0.31, 0.14)	0.4601
Model 2						
BMI	0.01 (0.00, 0.02)	0.0497	0.02 (0.01, 0.03)	<0.0001	-0.05 (-0.06, -0.04)	<0.0001
BMI categorical						
< = 18.5	Reference (0)		Reference (0)		Reference (0)	
>18.5, < = 25	0.04 (-0.19, 0.28)	0.7325	0.23 (0.03, 0.43)	0.0320	0.08 (-0.10, 0.26)	0.3824
>25, < = 30	0.08 (-0.18, 0.34)	0.5502	0.35 (0.14, 0.57)	0.0025	0.12 (-0.08, 0.32)	0.2458
>30	0.14 (-0.13, 0.41)	0.3166	0.51 (0.28, 0.74)	0.0001	-0.01 (-0.24, 0.21)	0.8991

For uric acid (mg/dl): survey-weighted coefficients (95% CI) p value

Crude model adjusted for: None

Model I adjusted for age, sex, race, hyperuricemia, hypertension, eGFR, DM, triglycerides, and direct HDL-cholesterol.

Model II adjusted for Model I + Education level, marital status, ratio of family income to poverty, work activity, recreational activity, smoking status, alcohol consumption level, triglycerides, creatinine, glucose, waist circumference, DBP, SBP, glycohemoglobin, LDL-cholesterol, doctor ever said you had arthritis, and minutes of sedentary activity.

Abbreviations: UA uric acid, BMI body mass index, SBP systolic blood pressure, DBP diastolic blood pressure, eGFR glomerular filtration rate

### Phosphorus affects the association between BMI and uric acid

Participants were grouped according to phosphorus concentration levels (T1: ≤3.3 mg/dl, T2: >3.3 mg/dl, <3.9 mg/dl, T3: ≥3.9 mg/dl), and we assessed the effect of phosphorus on the relationship between BMI and uric acid (Table 4). In the male population, the obese group (BMI>30) had a greater increase in uric acid levels per unit than the normal BMI group (BMI>18.5, < = 25) in all phosphorus groups. In adjusted model II, within the normal BMI group, the elevated UA levels were slightly lower in the high phosphorus group than in the low phosphorus group. However, within the other BMI groups, the results were reversed, with the high phosphorus group raising UA levels to a greater extent. In the female group and in the total population, UA levels were higher in the high phosphorus group than in the low phosphorus group in all BMI groups. In addition, the relationship between BMI and UA differed significantly by gender in response to phosphorus, with males and females in Model II having a greater increase in UA than females in most groups. In addition, phosphorus significantly altered the positive association between BMI and UA in most models.

**Table 4:** Phosphorus (mg/dl) affects the association between BMI and UA (mg/dl).

Exposure	T1 ≤ 3.3		T2 > 3.3, < 3.9		T3 ≥ 3.9		P interaction
	β (95%CI)	P value	β (95%CI)	P value	β (95%CI)	P value	
Male							
Crude model							0.0006
BMI categorical							
< = 18.5	Ref.		0.90 (0.12, 1.68)	0.0240	1.01 (0.30, 1.72)	0.0056	
>18.5, < = 25	1.15 (0.57, 1.73)	0.0001	1.08 (0.51, 1.66)	0.0002	1.08 (0.50, 1.66)	0.0003	
>25, < = 30	1.36 (0.78, 1.93)	<0.0001	1.50 (0.92, 2.07)	<0.0001	1.61 (1.03, 2.19)	<0.0001	
>30	1.81 (1.23, 2.38)	<0.0001	1.97 (1.39, 2.55)	<0.0001	2.11 (1.53, 2.69)	<0.0001	
Adjusted model I							0.0035
BMI categorical							
< = 18.5	Ref.		0.69 (0.16, 1.21)	0.0103	0.82 (0.35, 1.30)	0.0007	
>18.5, < = 25	0.81 (0.42, 1.20)	<0.0001	0.76 (0.37, 1.14)	0.0001	0.73 (0.35, 1.12)	0.0002	
>25, < = 30	0.91 (0.52, 1.29)	<0.0001	0.97 (0.58, 1.35)	<0.0001	0.92 (0.54, 1.31)	<0.0001	
>30	1.08 (0.69, 1.46)	<0.0001	1.18 (0.79, 1.57)	<0.0001	1.13 (0.74, 1.52)	<0.0001	
Adjusted model II							0.0023
BMI categorical							
< = 18.5	Ref.		0.70 (0.19, 1.22)	0.0077	0.87 (0.40, 1.34)	0.0003	
>18.5, < = 25	0.69 (0.31, 1.08)	0.0004	0.66 (0.28, 1.05)	0.0007	0.64 (0.26, 1.03)	0.0010	
>25, < = 30	0.70 (0.32, 1.09)	0.0003	0.77 (0.38, 1.16)	<0.0001	0.74 (0.35, 1.12)	0.0002	
>30	0.76 (0.36, 1.15)	0.0002	0.87 (0.47, 1.26)	<0.0001	0.83 (0.43, 1.23)	<0.0001	
Female							
Crude model							0.0861
BMI categorical							
< = 18.5	Ref.		0.49 (-0.40, 1.37)	0.2809	0.81 (0.01, 1.61)	0.0475	
>18.5, < = 25	0.94 (0.18, 1.70)	0.0155	1.01 (0.26, 1.77)	0.0084	1.10 (0.35, 1.86)	0.0040	
>25, < = 30	1.29 (0.53, 2.05)	0.0008	1.34 (0.59, 2.09)	0.0005	1.66 (0.91, 2.41)	<0.0001	
>30	1.87 (1.11, 2.62)	<0.0001	1.91 (1.16, 2.66)	<0.0001	2.18 (1.43, 2.93)	<0.0001	
Adjusted model I							0.0259
BMI categorical							
< = 18.5	Ref.		0.46 (-0.12, 1.04)	0.1206	0.65 (0.12, 1.17)	0.0158	
>18.5, < = 25	0.69 (0.19, 1.19)	0.0070	0.71 (0.21, 1.20)	0.0050	0.83 (0.33, 1.32)	0.0011	
>25, < = 30	0.88 (0.38, 1.38)	0.0006	0.91 (0.42, 1.40)	0.0003	1.04 (0.54, 1.53)	<0.0001	
>30	1.19 (0.70, 1.69)	<0.0001	1.19 (0.70, 1.68)	<0.0001	1.21 (0.72, 1.71)	<0.0001	
Adjusted model II							0.0437
BMI categorical							
< = 18.5	Ref.		0.46 (-0.12, 1.03)	0.1196	0.62 (0.10, 1.14)	0.0189	
>18.5, < = 25	0.57 (0.07, 1.06)	0.0251	0.58 (0.09, 1.07)	0.0195	0.69 (0.21, 1.18)	0.0054	
>25, < = 30	0.63 (0.14, 1.13)	0.0125	0.66 (0.17, 1.15)	0.0087	0.78 (0.29, 1.27)	0.0019	
>30	0.77 (0.27, 1.27)	0.0024	0.78 (0.28, 1.27)	0.0022	0.79 (0.30, 1.29)	0.0018	
Total							
Crude model							<0.0001
BMI categorical							
< = 18.5	Ref.		0.68 (0.10, 1.25)	0.0207	0.88 (0.37, 1.39)	0.0007	
>18.5, < = 25	1.08 (0.62, 1.54)	<0.0001	1.05 (0.60, 1.51)	<0.0001	1.11 (0.65, 1.56)	<0.0001	
>25, < = 30	1.34 (0.88, 1.79)	<0.0001	1.43 (0.97, 1.88)	<0.0001	1.64 (1.19, 2.10)	<0.0001	
>30	1.83 (1.38, 2.29)	<0.0001	1.94 (1.49, 2.40)	<0.0001	2.16 (1.71, 2.62)	<0.0001	
Adjusted model I							0.0052
BMI categorical							
< = 18.5	Ref.		0.55 (0.17, 0.93)	0.0049	0.70 (0.36, 1.04)	<0.0001	
>18.5, < = 25	0.77 (0.46, 1.07)	<0.0001	0.72 (0.42, 1.03)	<0.0001	0.79 (0.48, 1.09)	<0.0001	
>25, < = 30	0.90 (0.59, 1.20)	<0.0001	0.93 (0.63, 1.24)	<0.0001	0.98 (0.68, 1.29)	<0.0001	
>30	1.11 (0.81, 1.42)	<0.0001	1.18 (0.87, 1.48)	<0.0001	1.18 (0.87, 1.48)	<0.0001	
Adjusted model II							0.0075
BMI categorical							
< = 18.5	Ref.		0.55 (0.18, 0.93)	0.0041	0.69 (0.36, 1.03)	<0.0001	
>18.5, < = 25	0.64 (0.34, 0.94)	<0.0001	0.61 (0.31, 0.91)	<0.0001	0.67 (0.37, 0.98)	<0.0001	
>25, < = 30	0.66 (0.36, 0.97)	<0.0001	0.71 (0.41, 1.01)	<0.0001	0.76 (0.45, 1.06)	<0.0001	
>30	0.75 (0.44, 1.05)	<0.0001	0.81 (0.50, 1.12)	<0.0001	0.82 (0.51, 1.13)	<0.0001	

Crude model adjusted for: None

Model I adjusted for age, sex, race, hyperuricemia, hypertension, eGFR, DM, triglycerides, and direct HDL-cholesterol.

Model II adjusted for Model I + Education level, marital status, ratio of family income to poverty, work activity, recreational activity, smoking status, alcohol consumption level, triglycerides, creatinine, glucose, waist circumference, DBP, SBP, glycohemoglobin, LDL-cholesterol, doctor ever said you had arthritis, and minutes of sedentary activity.

Convert to dietary intake Vitamin D trisection range: ≤3.3 mg/dl, 3.4–3.8 mg/dl, ≥3.9 mg/dl

Results in table: β (95% CI) P value

Abbreviations: UA uric acid, BMI body mass index, SBP systolic blood pressure, DBP diastolic blood pressure, eGFR glomerular filtration rate

### Linear correlation between BMI and UA in hierarchical analysis

We conducted a stratified analysis based on age, gender, ethnicity, educational attainment, marital status, household income to poverty ratio, work activity and recreational activity. The results of the stratified analysis are shown in Table 5, showing broad consistency and stability across all strata, with a positive correlation between BMI and UA (p<0.05). The results of the subgroup analysis were highly consistent with the multivariable linear regression analysis results.

**Table 5:** Linear correlation between BMI and UA in hierarchical analysis.

Stratified variables	N	β (95%CI) for UA	P value
Age (years)			
20–39	3540	0.05 (0.05, 0.06)	<0.0001
40–58	3475	0.05 (0.04, 0.06)	<0.0001
59–80	3771	0.06 (0.05, 0.07)	<0.0001
Sex			
male	5404	0.06 (0.06, 0.07)	<0.0001
female	5382	0.06 (0.05, 0.06)	<0.0001
Race			
Mexican American	1599	0.05 (0.04, 0.06)	<0.0001
Other Hispanic	1106	0.07 (0.05, 0.08)	<0.0001
Non-Hispanic White	4794	0.07 (0.06, 0.07)	<0.0001
Non-Hispanic Black	2093	0.04 (0.03, 0.05)	<0.0001
Other Race—Including Multi-Racial	1194	0.07 (0.05, 0.08)	<0.0001
Education level			
≤High school	3408	0.04 (0.04, 0.05)	<0.0001
>High school	7378	0.06 (0.05, 0.06)	<0.0001
Marital status			
Married	5617	0.06 (0.06, 0.07)	<0.0001
Widowed	781	0.05 (0.03, 0.07)	<0.0001
Divorced	1196	0.05 (0.04, 0.06)	<0.0001
Separated	363	0.04 (0.02, 0.06)	0.0001
Never married	1952	0.05 (0.04, 0.06)	<0.0001
Living with partner	877	0.05 (0.04, 0.06)	<0.0001
Ratio of family income to poverty			
< = 1	2244	0.04 (0.04, 0.05)	<0.0001
1–3	4497	0.05 (0.05, 0.06)	<0.0001
≥3	4045	0.07 (0.06, 0.07)	<0.0001
Work activity			
no	6139	0.05 (0.05, 0.06)	<0.0001
both	1765	0.06 (0.05, 0.07)	<0.0001
moderate	2450	0.06 (0.05, 0.07)	<0.0001
vigorous	432	0.04 (0.02, 0.06)	0.0003
Recreational activity			
no	5476	0.05 (0.05, 0.06)	<0.0001
both	1598	0.06 (0.05, 0.07)	<0.0001
moderate	2873	0.06 (0.05, 0.06)	<0.0001
vigorous	839	0.06 (0.05, 0.08)	<0.0001

## Discussion

Analyzing data from the adult population of the United States from the NHANES which is national and representative, this study showed that there was a weak but significant correlation between BMI and serum uric acid in females. Additionally, the prevalence of hyperuricemia within the group was higher with higher BMI values. In addition, an interaction between phosphorus and BMI and SUA was found, suggesting that the interaction between phosphorus exposure and BMI is more dangerous than the sum of the individual effects.

Obesity is a risk factor for the development of hyperuricemia [28]. Hyperuricemia in obesity is mainly attributed to increased purine intake and impaired renal clearance of UA [29, 30]. Recently, many studies have shown a significant correlation between BMI and uric acid. There is a study proposing that high serum uric acid is the result rather than the cause of elevated BMI regarding the relationship between uric acid and BMI [31]. Several studies have confirmed a positive correlation between BMI and uric acid, whether in children, adults, or women, and BMI was significantly associated with uric acid, as was the case in people with type 2 diabetes [4, 32, 33]. Chonin Cheang, who studied the relationship between obesity and uric acid, found that the prevalence of hyperuricemia among obese patients was as high as 69.8% and that the prevalence of HUA increased with increasing BMI [34]. Our study also obtained results on the effect of sex on the relationship between BMI and uric acid. Our findings suggest that beta values are greater in men than in women for the same BMI. Epidemiological studies, which initially emphasized the urinary effects of estrogen, showed elevated serum uric acid (sUA) levels in postmenopausal women [35], and estrogen therapy reduces serum uric acid concentrations and increases uric acid excretion [36]. Children who were overweight or obese were more likely to have higher uric acid levels [37]. In addition, sUA levels increased in children with increased weight and, conversely, sUA levels decreased in children with decreased weight [38]. However, some studies contradict our findings. The research conducted by Hui Zhou [18] found that BMI and uric acid have a U-shaped relationship, but the study is limited to coastal areas, so dietary habits are is special. The consumption of marine products, including marine fish, shellfish, and shrimp, has a greater impact on uric acid. Rasika C [16] found that serum uric acid increased proportionally with increasing BMI, but there was no statistical significance. His experimental research object was limited to pregnant women, the study time was limited, and the sample size of this study was small. The study of Laughon S.K. [15] proved that although uric acid is closely related to BMI, it has nothing to do with BMI, whose reference population was also limited to women. Compared with the above studies, our study has a larger sample size and a wider population.

How to explain our results that BMI>25 is positively correlated with uric acid but BMI 18.5–25 is not? 18.5–25 is the BMI range of normal people [18], and there is no research on the relationship between BMI and uric acid within the normal range of BMI. Patients with BMI>25 are overweight patients [18]. The mechanism that BMI is proportional to uric acid when BMI>25 is the mechanism by which overweight leads to high uric acid. Although the exact mechanism of serum uric acid elevation in obesity has not been fully elucidated, several reasons have been proposed. Xanthine oxidoreductase activity in adipose tissue thereby grades hypoxanthine and xanthine to uric acid [39]. Honggang Wang [40] believes that this is probably because those who are obese accumulate excessive amounts of fat, which can produce and secrete uric acid. This may confirm our results that BMI≥25 is significantly associated with uric acid, whereas BMI 18.5–25 is not.

Bedir A [41] and Fruehwald-Schultes B [42] found that serum uric acid concentration was independently associated with serum leptin concentration, which suggested that leptin may be a pathogenic factor of hyperuricemia in obese patients. In addition, the increase in uric acid in obese patients may also be influenced by differences in lipid distribution. Obese patients have increased visceral fat, and when the accumulation of visceral fat increases, the uric acid concentration also increases correspondingly [21]. Visceral fat accumulation induces a large flow of plasma free fatty acids into the active portal vein and hepatic portal vein, which stimulates triglyceride synthesis and subsequently leads to a surge in uric acid production by activating the uric acid synthesis pathway [26]. BMI, hyperinsulinemia and insulin resistance are independent risk factors for hyperuricemia, and the occurrence of hyperuricemia in obese people may be related to hyperinsulinemia or insulin resistance [43]. Elevated serum uric acid in obese patients is also associated with insulin resistance, who often have insulin resistance [19]. Insulin resistance may be a cause of high serum uric acid levels, as the presence of hyperinsulinemia may reduce uric acid excretion, leading to uric acid metabolism disorders [20]. Data from the H Vuorinen-Markkola study suggest that hyperuricemia is an intrinsic component of metabolic syndrome and may also serve as a simple marker of insulin resistance [44]. In addition, as mentioned in the introduction, obesity is a basic risk factor for the onset and development of insulin resistance, and obese people tend to have insulin resistance [19]. UA can directly inhibit the insulin signaling pathway by promoting the binding of human vascular smooth muscle cells to insulin receptors by pyrophosphatase, which enhances the mechanism of insulin resistance in turn [45]. In contrast, when the BMI range is 18.5–25, the reason why there is no significant correlation between BMI and uric acid may be because people in the normal BMI range have less visceral fat and no insulin resistance. Supersaturation of uric acid increases with BMI [46].

Phosphorus has extracellular and intracellular distributions, is a structural component of bones and teeth as well as DNA/RNA, and makes lipid membranes and circulating lipoproteins bipolar. Metabolically, phosphorus functions in key pathways to generate and store energy in phosphate bonds (ATP), buffer blood, regulate gene transcription, activate enzyme catalysis, and enable signal transduction in regulatory pathways affecting various organ functions from renal excretion to immune responses [47]. In our study, an interaction of phosphorus on BMI and SUA was found, suggesting that the interaction of phosphorus exposure with BMI is more dangerous than the sum of the individual effects.

Hyperphosphatemia is caused by a reduced renal filtration rate, hyperparathyroidism, hyperthyroidism, increased P load, antacids, diet and acute destruction of any tissue. Regarding the relationship between phosphorus and BMI, studies have shown that patients with hyperphosphatemia have higher albumin levels and higher protein intake, as well as a higher BMI [9]. An increase in plasma uric acid is associated with a slight decrease in plasma phosphorus [48]. phosphorus levels were significantly associated with prognostic factors related to renal insufficiency, and patients with hyperphosphatemia had higher serum uric acid than those without hyperphosphatemia [25]. Shuto et al. found that phosphorus loading contributed sharply to endothelial dysfunction by increasing ROS production and decreasing nitric oxide [49], which causes insulin resistance and leads to disorders of uric acid metabolism [20]. Shin JY showed that phosphorus levels were positively correlated with serum uric acid in both sexes and that phosphorus-induced ROS overload resulted in insulin resistance [50].

Our study has some advantages. First, this study revealed for the first time the interaction of phosphatemia on BMI and uric acid, which may have important implications for controlling the intake of phosphatemia content in BMI-high populations. Second, most of the potential distractions and effect modifiers were adjusted. Third, our data sample is much larger than in previous studies.

However, our study has several limitations. First, due to the cross-sectional design, we were unable to demonstrate causality or directionality. Second, even after multiple adjustments, the results may be affected by some other variable that cannot be measured. Finally, the study included only US residents although large quantities of samples were included, so population differences should be considered when inferring the relationship between BMI and uric acid in other populations. As a result, our findings also require a reasonable multisample multicenter controlled trial.

## Conclusion

Our results showed a significant association between BMI and uric acid, with those with a higher BMI at a higher risk of developing hyperuricemia, suggesting that we should closely monitor uric acid levels in obese individuals in clinical practice. Phosphorus and BMI had an interaction in elevating UA and should be noted as an indicators of phosphorus in clinical practice.

## Supporting information

S1 Table Standardized β of all covariates (predictors) in the fully adjusted model for prediction of uric acid. (TIFF)

S2 Table Analysis of the threshold effect of BMI on the prevalence of uric acid (UA). (TIFF)
