# Correction: Phosphorus modifies the association between body mass index and uric acid: Results from NHANES 2007–2018

**DOI:** 10.1371/journal.pone.0323008

**Published:** 2025-04-22

**Authors:** The PLOS One Staff

There are errors in the author affiliations. The correct affiliations are as follows:

Yue Chen^1,2^, Jing Luo^1,3^, Xiao-Man Ma^1,3^, Xiang-Ping He^1,4^, Wan-Lin Zhang^1,5^, Shao-Yong Wu^1,4^, Xiao-Chun Mo^1,3^, Wei-Chao Huang^1,6^, Xu-Guang Guo^1,3,7^

1 Department of Clinical Laboratory Medicine, Guangdong Provincial Key Laboratory of Major Obstetric Diseases, Guangdong Provincial Clinical Research Center for Obstetrics and Gynecology, The Third Affiliated Hospital, Guangzhou Medical University, Guangzhou, China, 2 Department of Anesthesiology, The Second Clinical School of Guangzhou Medical University, Guangzhou, China, 3 Department of Clinical Medicine, The Third Clinical School of Guangzhou Medical University, Guangzhou, China, 4 Department of Clinical Medicine, The Chinese and Western Clinical School of Guangzhou Medical University, Guangzhou, China, 5 Department of Clinical Medicine, The First Clinical School of Guangzhou Medical University, Guangzhou, China, 6 Department of Clinical Medicine, The Second Clinical School of Guangzhou Medical University, Guangzhou, China, 7 Guangzhou Key Laboratory for Clinical Rapid Diagnosis and Early Warning of Infectious Diseases, King Med School of Laboratory Medicine, Guangzhou Medical University, Guangzhou, China.

The publisher apologizes for the error.
