# Supplementary figures and images for: Phosphorus modifies the association between body mass index and uric acid: Results from NHANES 2007–2018
Source: PLoS One. 2024 Oct 10;19(10):e0306383. doi: 10.1371/journal.pone.0306383 (PMC11469615; doi:10.1371/journal.pone.0306383)

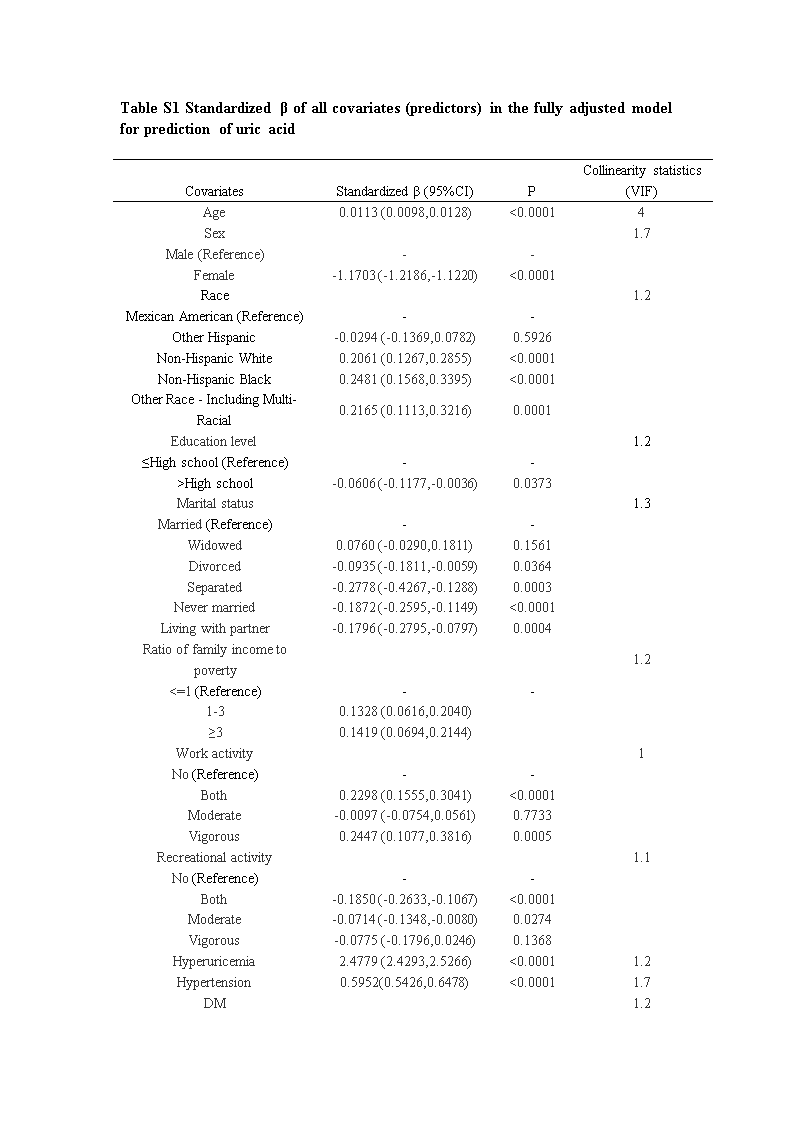

Supplement: S1 Table — (TIFF) [file pone.0306383.s001.tiff]

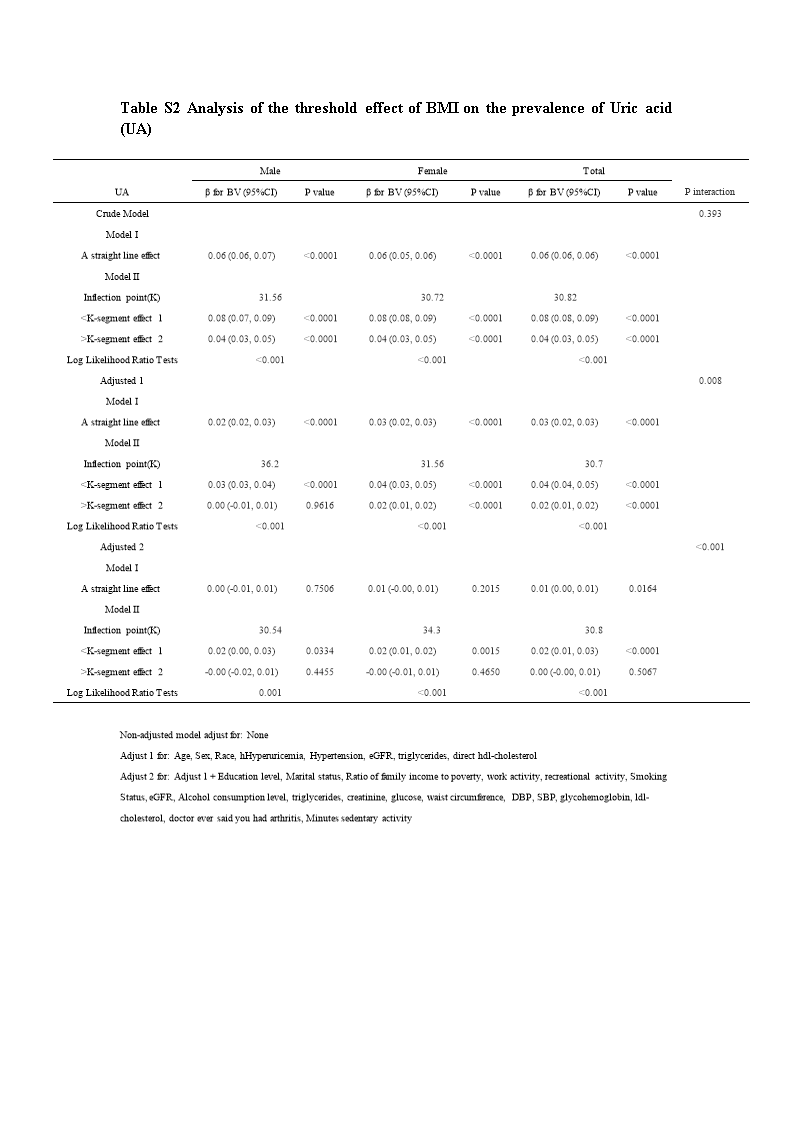

Supplement: S2 Table — (TIFF) [file pone.0306383.s002.tiff]
